# An ancient oxidase lost in vertebrates promotes extreme stress tolerance in an emerging cnidarian model for ecology, evolution and biomedicine

**DOI:** 10.1242/jeb.252244

**Published:** 2026-05-26

**Authors:** Giulia S. Rossi, Sanjana Venkatesh, Allison E. McDonald, Alexander G. Little

**Affiliations:** ^1^ Department of Biology, McMaster University, Hamilton, Ontario, Canada, L8S 4L8; ^2^ Department of Biology, Wilfrid Laurier University, Waterloo, Ontario, Canada, N2L 3C5

**Keywords:** Hypoxia, Hydrogen sulphide, Thermal tolerance, Oxidative stress, Mitochondrial respiration, Metabolic plasticity

## Abstract

Aerobic respiration underpins animal performance, yet the mitochondrial electron transport system is often treated as a single, vertebrate-centric blueprint. This view overlooks alternative oxidase (AOX), an enzyme that allows electrons to bypass complexes III and IV, partially uncoupling oxidative phosphorylation. Although AOX is well studied in plants for its role in stress tolerance, its presence in animals was recognized only recently, leaving its contribution to metabolic flexibility underappreciated. Here, we used an emerging ecological–evolutionary–developmental biology (eco-evo-devo) and biomedical model, *Nematostella vectensis*, to test the hypothesis that AOX supports stress tolerance by bypassing complex IV (cytochrome *c* oxidase; COX) during hydrogen sulphide (H_2_S) exposure and by mitigating oxidative damage under hypoxia and heat stress via reduced reactive oxygen species production. We found that anemones upregulated AOX protein expression after H_2_S exposure and exhibited cyanide-resistant respiration, consistent with continued electron flow despite COX inhibition. Behavioural assays showed that AOX inhibition increased sensitivity to H_2_S, declining oxygen and heat, whereas biochemical assays revealed that AOX inhibition led to elevated lipid peroxidation and protein carbonylation with hypoxia and heat exposure. Together, these results establish AOX as a critical yet overlooked mechanism of metabolic flexibility that buffers aerobic metabolism against multiple stressors, challenging textbook portrayals of conserved mitochondrial function and offering new perspectives on how animals persist in a rapidly changing world.

## Teaser

**Summary:** Alternative oxidase (AOX), a mitochondrial enzyme only recently identified in animals, plays a central role in buffering against extreme stress, including toxic levels of hydrogen sulphide, hypoxia and heat.

## INTRODUCTION

Aerobic respiration is the primary source of energy (ATP) for most animals, powering the cellular processes that underpin life. At its core lies the mitochondrion, which converts nutrients into ATP through oxidative phosphorylation (Schultz and Chan, 2001). Animal mitochondria are highly sensitive to environmental stress, and stress-induced disruptions to mitochondrial function can have major consequences for organismal performance and fitness (Sokolova, 2018; Koch et al., 2021). Today, anthropogenic activities are intensifying stressors that can disrupt mitochondrial function by altering biochemical reaction rates (e.g. warming; Abele et al., 2002) and substrate availability (e.g. hypoxia; Adzigbli et al., 2022), undermining energy balance (e.g. pollution; Padmini and Rani, 2011) and promoting reactive oxygen species (ROS) production (e.g. UV radiation; Heck et al., 2003; Kazerouni et al., 2016). Understanding how perturbations in mitochondrial function and aerobic metabolism can shape population- and species-level vulnerability to climate change has become a cornerstone of ecophysiology and conservation biology in recent decades.

Despite textbook portrayals of mitochondrial physiology as relatively conserved across the animal kingdom, emerging evidence has revealed a surprising degree of diversity. A historical focus on the ‘conventional’ vertebrate electron transport pathway – where electrons from metabolic substrates pass through four respiratory complexes (I–IV) to create a proton gradient that drives ATP synthesis via complex V – fundamentally underestimates the capacity for metabolic flexibility among animals. This blind spot may limit our ability to extrapolate from organismal-level physiology to population- or species-level resilience to global climate change. More broadly, it could also constrain our understanding of other important ecological and physiological phenomena, from species range expansions and biological invasions (Bremer et al., 2021) to the mitigation of metabolic diseases (Viscomi et al., 2023). Over the past several decades, it has become clear that many animals possess alternative electron transport components that give rise to a branched, rather than linear, electron transfer process. For example, many sessile aquatic invertebrates express an alternative to complex I (alternative NADH dehydrogenase; NDH2) and an alternative terminal oxidase (alternative oxidase; AOX), which provide distinct entry and exit points for electrons, respectively (McDonald and Gospodaryov, 2019). These alternative respiratory pathways confer metabolic flexibility that may help buffer aerobic processes against disruptions caused by environmental stress.

**Fig. 1.**
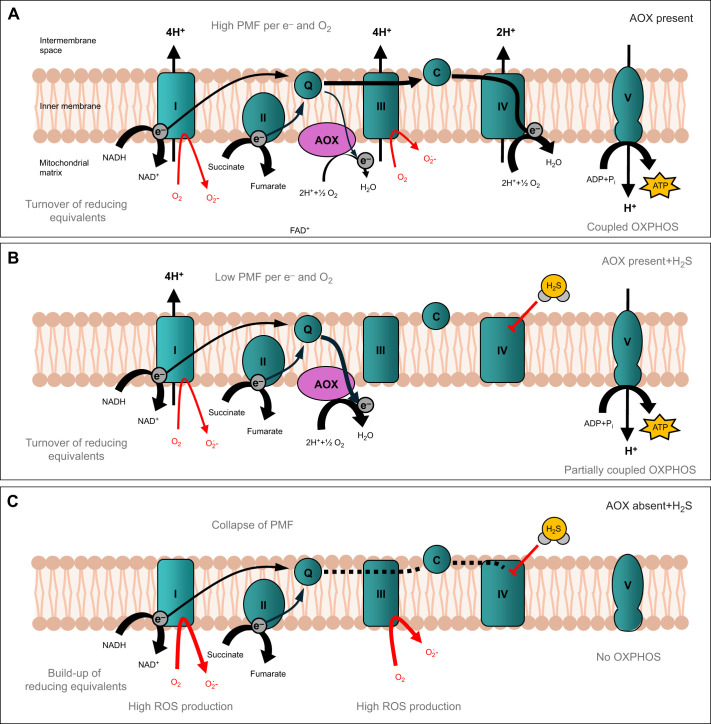
**Electron transport system of *Nematostella vectensis*.** (A) The mitochondrial ETS showing the position of AOX, which introduces a branch point at the ubiquinone (Q) pool. Complexes I, III and IV move protons across the inner mitochondrial membrane into the intermembrane space, generating the proton motive force (PMF) that drives ATP synthesis via ATP synthase (complex V). In contrast, the AOX pathway bypasses the proton-pumping activities of complexes III and IV, thereby reducing ATP synthesis capacity. (B) The ETS of an AOX-possessing animal under complete COX inhibition by H_2_S. Electron flow is redirected through AOX, resulting in a lower PMF per electron and oxygen molecule and resulting in only partially coupled oxidative phosphorylation (OXPHOS). (C) The ETS of an animal lacking AOX with COX inhibited by H_2_S, illustrating the collapse of the PMF, accumulation of reducing equivalents, elevated ROS production and loss of OXPHOS.

AOX is an inner mitochondrial membrane protein that introduces a branch point at the ubiquinone pool, diverting electrons away from complexes III and IV, and reducing oxygen (O_2_) to water (H_2_O) (Vanlerberghe and McIntosh, 1997) (Fig. 1A). Unlike its terminal oxidase counterpart, cytochrome *c* oxidase (complex IV; COX), AOX does not pump protons across the inner mitochondrial membrane. Thus, electron flow through AOX bypasses the proton-pumping activities of complexes III and IV, effectively uncoupling oxidative phosphorylation and dissipating energy as heat (McIntosh, 1994; McDonald et al., 2018). Although the presence of AOX is well established in plants, fungi and protists, its presence in animals was only discovered in 2004 (McDonald and Vanlerberghe, 2004). Though AOX is considered evolutionarily ancestral to animals, it has been secondarily lost in several major lineages, most notably vertebrates (Jacobs and Ballard, 2022). Despite its broad phylogenetic distribution, however, the functional role(s) of AOX in animals remains poorly understood.

In plants, AOX has been widely implicated in promoting tolerance to a broad range of environmental stressors, including cold (Erdal et al., 2015), heat (Borovik and Grabelnych, 2018), drought (Vanlerberghe et al., 2016), infection (Simons et al., 1999), UV radiation (Xu et al., 2024), hypoxia (Jayawardhane et al., 2020), salinity (Manbir et al., 2022) and pesticide exposure (Alimova et al., 2024). This broad tolerance is thought to arise from its promotion of metabolic flexibility, with a central hypothesis suggesting that its energy-dissipating nature moderates mitochondrial membrane potential and dampens ROS production (Purvis and Shewfelt, 1993).

Only a handful of studies have examined AOX expression in animals, but it appears that its role in stress tolerance is conserved. For example, AOX protein expression increases in the copepod *Tigriopus californicus* under cold and heat stress (Tward et al., 2019), and transcript levels increase in response to hypoxia and anoxia in the bivalves *Crassostrea gigas* and *Diplodon chilensis* (Sussarellu et al., 2012; Yusseppone et al., 2018). Additionally, AOX also promoted developmental success of the ascidian *Ciona intestinalis* when exposed to hydrogen sulphide (H_2_S), a potent inhibitor of COX (Bremer et al., 2021). By allowing electron transport and potentially some ATP production to continue when COX is inhibited, AOX provides a key mechanism of tolerance to both H_2_S and cyanide, which also targets COX (Fig. 1B). Accordingly, AOX has been identified in several invertebrate species (Weaver and McDonald, 2023), including some known to burrow in sulphidic sediments. Beyond its role in environmental stress tolerance, studies using heterologous AOX expression in biomedical models have begun exploring its potential in mitigating human disease (Viscomi et al., 2023). Expression of *C. intestinalis* AOX in mice reduced the development of smoke-induced emphysema by decreasing mitochondrial superoxide production and preserving cell viability (Giordano et al., 2019). Despite these insights, the functional importance of animal AOX remains poorly understood but presents a powerful opportunity to uncover insights into a mechanism of stress tolerance across diverse animal lineages.

We used the starlet sea anemone (*Nematostella vectensis*) to investigate the functional role of AOX in promoting tolerance to three environmental stressors that are intensifying with global climate change: H_2_S, hypoxia and warming. Native to the Atlantic coast of North America, *N. vectensis* inhabits the hypoxic and H_2_S-rich sediments of coastal salt marshes, lagoons and mudflats. It is a powerful model for this work because it possesses an *AOX* gene (Weaver and McDonald, 2023) and it exhibits exceptional stress tolerance (Reitzel et al., 2008a). Beyond its relevance to climate change resilience, *N. vectensis* is also emerging as a valuable model in both ecological–evolutionary–developmental biology (eco-evo-devo) and biomedical research. For example, human activities have facilitated recent *N. vectensis* invasions along the Pacific coast of North America and in the UK, offering opportunities to trace their invasion success (Reitzel et al., 2008b; Darling et al., 2009). In another invasive invertebrate, *C. intestinalis*, AOX has been posited as a mediator of invasion potential owing to its role in enhancing environmental stress tolerance (Bremer et al., 2021). From a biomedical perspective, *N. vectensis* has also proven valuable in studies on regeneration and wound healing (Röttinger, 2021), processes that are notoriously ROS-regulated (Hunt et al., 2024). However, whether AOX plays a role in its extraordinary regenerative potential has not been explored.

We tested the hypothesis that AOX supports tolerance to ecologically relevant stressors in *N. vectensis* either by circumventing COX inhibition (during H_2_S exposure) or by dampening ROS production to protect against oxidative damage (during hypoxia or heat exposure). We first evaluated changes in AOX protein expression following brief exposures to H_2_S, hypoxia or heat. Using pharmacological inhibitors of AOX (salicylhydroxamic acid; SHAM) and COX (potassium cyanide; KCN), we then assessed the relative impacts of AOX, COX and combined AOX/COX inhibition on whole-animal O_2_ consumption under control and stressor conditions. As another measure of whole-animal performance, we conducted behavioural assays with inhibitor-treated anemones to link AOX activity to enhanced tolerance of sulphidic, hypoxic and thermal extremes. Finally, we assessed markers of oxidative damage (lipid peroxidation, protein carbonylation) in anemones exposed to hypoxia or heat, with and without AOX inhibition. Sitting at the intersection of models for ecology, evolution and regenerative medicine, mechanistic insights into the role of AOX in *N. vectensis* promise broad, transdisciplinary applications.

## MATERIALS AND METHODS

### Experimental animals

We collected adult starlet sea anemones (*Nematostella vectensis* Stephenson 1935) from coastal sediments at Grand Desert Beach in the Chezzetcook Inlet, Nova Scotia, during August 2023 and 2024, following a collection protocol previously described (Stefanik et al., 2013). Briefly, soft sediment was scooped into shallow trays and allowed to settle, after which anemones were collected using a transfer pipette as they emerged from the substrate with their tentacles extended. All anemones were transported to McMaster University, Ontario, Canada, where they were maintained in Petri dishes (∼150 ml water, 21°C, 30‰ salinity) under a 12 h:12 h light:dark photoperiod. We performed water changes twice weekly and fed anemones live *Artemia* sp. nauplii three times weekly.

### AOX western blotting

To confirm whether *N. vectensis* expresses AOX protein and modulates its expression in response to environmental stressors, we exposed anemones to one of four treatments for 24 h: (1) control conditions (21°C, 30‰ salinity), (2) elevated H_2_S, (3) low O_2_ or (4) elevated temperature (31°C) (*n*=3 per treatment). For the elevated H_2_S treatment, anemones were initially exposed 485.4 μmol l^−1^ H_2_S, prepared from Na_2_S·9H_2_O (Sigma-Aldrich Canada, Oakville, Canada) dissolved in seawater (30‰ salinity). The pH of the exposure water was adjusted to 7 to ensure that roughly 60% of total sulphides in solution (H_2_S, HS^−^, S^2–^) were present as toxic H_2_S (Broderius and Smith, 1977). Because H_2_S is unstable, some loss due to oxidation and/or volatilization was unavoidable. Thus, we conducted the exposure in a sealed 15 ml Falcon tube to minimize H_2_S loss (5.6% per hour) and measured total sulphides at four time points throughout the 24-h exposure colorimetrically using the Methylene Blue method (Cline, 1969). We calculated the H_2_S concentration based on water pH, temperature and total sulphide concentration as described by Broderius and Smith (1977). Low O_2_ exposure was achieved by sealing individual anemones in the wells of a microplate (1.7 ml water; 30‰ salinity) for 24 h. O_2_ levels were continuously monitored using the Loligo^®^ Microplate Respirometry System (Loligo^®^ Systems, SY25040), with air saturation declining from 100% to 0% after 13 h and remaining anoxic for the remainder of the exposure. Consistent temperatures (21°C, 31°C) were maintained using a Fisherbrand™ Isotemp™ incubator. Survival was 100% across all treatments, as confirmed by the presence of extended tentacles following overnight recovery in clean water. Following the 24-h exposures, total protein was extracted from each anemone by homogenizing the whole animal in tissue extraction buffer (100 mmol l^−1^ TRIS, 100 mmol l^−1^ NaCl, 5 mmol l^−1^ EDTA, 1 mmol l^−1^ PMSF). Homogenates were then centrifuged at 15,000 ***g*** for 15 min at 4°C. The resulting supernatant was collected and used to estimate protein concentration via the Bradford protein assay (Bradford, 1976), then stored at −80°C until western blot analysis.

The supernatant was mixed with loading buffer (2× Laemmli Sample Buffer with 5% β-mercaptoethanol; Bio-Rad Laboratories, Hercules, CA, USA) and heated for 5 min at 95°C. Equal amounts of protein from each sample (13 μg) were loaded into 10% Mini-PROTEAN^®^ TGX™ Precast Protein Gels (Bio-Rad). A total of 5 μl of Precision Plus Protein Western C Blotting Standards was loaded in the first lane of the gel. Proteins were subsequently transferred to nitrocellulose membranes using the Trans-Blot Turbo Transfer System (Bio-Rad) at 25 V for 3 min. After the transfer, the membrane was visualized using a VersaDoc imaging system (Bio-Rad) to confirm consistent protein loading. We then incubated the membranes in EveryBlot Blocking Buffer (Bio-Rad) for 5 min at room temperature with gentle agitation. Next, we incubated the membranes in primary antibody (Plant AOX1/2, catalogue no. ABIN3197483, Agrisera Antibodies) diluted 1:5000 in blocking buffer for 1 h at room temperature with gentle agitation. This primary antibody has previously been used to detect animal AOX in animal tissue (*T. californicus*; Tward et al., 2019). The membranes were then washed five times (5 min each) in TBST (0.05% Tween-20 in 1X TBS). We then incubated the membranes in secondary antibodies: a goat anti rabbit IgG (catalogue no. ABIN101988, Agrisera Antibodies, dilution 1:10,000) and a Precision Protein StrepTactin HRP Conjugate (Bio-Rad; 1:10,000), both diluted in blocking buffer. Membranes were then washed six times in TBST for 5 min each with gentle agitation. The blot was developed using a 1:1 mixture of luminol/enhancer and peroxide buffer solution from the Immun Star Western C Chemiluminescent kit (Bio-Rad) for 5 min. We then placed the membranes in the VersaDoc imagine system and imaged the chemiluminescent signal.

### Whole-animal respirometry

We used closed-system respirometry to assess how the rate of O_2_ consumption in *N. vectensis* is influenced by the AOX and COX electron transport pathways (*N*=123; 13.7±0.8 mg, mean±s.e.m). Individual anemones were placed into single wells of the Loligo^®^ Microplate Respirometry System and allowed a 20- to 30-min adjustment period to resume an extended tentacle posture. We then exposed the anemones to one of the following drug exposures: 5 mmol l^−1^ potassium cyanide (KCN) to inhibit COX, 5 mmol l^−1^ salicylhydroxamic acid (SHAM) to inhibit AOX, both inhibitors simultaneously, or a vehicle control. We chose relatively high inhibitor concentrations because whole-animal exposures typically require higher doses than isolated mitochondrial or *in vitro* assays for complete enzyme inhibition. Owing to the low solubility of SHAM in water, this treatment was prepared in dimethyl sulfoxide (DMSO) and added to a final concentration of 1% (v/v) per well. The vehicle control group was exposed to 1% DMSO without inhibitors, and 1% DMSO was also added to the KCN treatment group to match solvent exposure. Each plate also contained at least one blank well for every drug treatment, prepared identically but without an anemone, to correct for background bacterial respiration. Immediately after drug addition, plates were sealed with an O_2_-impermeable adhesive film (Whatman™ Uniseal), and O_2_ concentrations were recorded at 15-s intervals. To assess AOX and COX function under environmental stress, respirometry was performed under three conditions: standard temperature (21°C, *n*=9–13 per group), high temperature (31°C, *n*=8–11) and hypoxia (21°C, ∼45% air saturation, *n*=9–12). Temperature was controlled using a Fisherbrand™ Isotemp™ incubator, while hypoxia was induced by bubbling N_2_ through the experimental water prior to assays. In 21°C and 31°C trials, O_2_ levels were recorded for approximately 3 h, during which time air saturation declined from 100.0±0.1% to 83.0±2.2% (20.3±0.0 to 17.2±0.5 kPa) and 99.9±0.0% to 89.5±2.4% (20.3±0.01 to 18.2±0.5 kPa) at 21°C and 31°C, respectively. All experiments were performed in the dark on a shaking platform to ensure sufficient water mixing. The hypoxia trials were carried out for approximately 8 h, during which air saturation declined from 43.8±1.1% to 25.0±2.2% (9.1±0.2 to 5.2±0.5 kPa). Given the longer measurement period under hypoxic conditions, O_2_ levels were measured at 30-s intervals. Anemones were fasted for at least 24 h and weighed to the nearest milligram before experimentation, with O_2_ consumption rates subsequently normalized to wet tissue mass. Microplates were calibrated weekly.

### Stressor sensitivity assay

We used a behavioural assay to evaluate how the inhibition of COX or AOX influences the sensitivity of *N. vectensis* to various environmental stressors. Sensitivity was determined as the proportion of anemones that remained responsive to a gentle prod (indicated by visible tentacle movement) during exposure to increasing H_2_S concentrations, increasing temperatures or decreasing O_2_ levels. Individual anemones were placed into the wells of perforated six-well plates, which were suspended in glass containers (20×12×7 cm) filled with 150 ml of water (30‰ salinity). One container was treated with 5 mmol l^−1^ KCN to inhibit COX, another with 5 mmol l^−1^ SHAM to inhibit AOX, and a third served as a vehicle control, receiving 1% DMSO without inhibitors. To ensure consistent solvent exposure, 1% DMSO was also added to the KCN treatment group.

To expose anemones to increasing H_2_S concentrations, we prepared new glass containers with 150 ml of water containing the respective drug treatments and added varying amounts of a 10 mmol l^−1^ sulphide stock solution, prepared using Na_2_S·9H_2_O (pH=7). At 10-min intervals, the perforated six-well plates containing the anemones were carefully transferred from one glass container to the next, exposing anemones to progressively higher H₂S concentrations. Over the course of the experiment, which lasted approximately 90 min, H_2_S levels increased from 0 to 3 mmol l^−1^ across eight sequential container transitions. Anemone responsiveness was recorded immediately prior to each transition. We measured the concentration of total sulphides at the start and end of each 10-min interval colorimetrically to evaluate H_2_S loss (Cline, 1969). On average, 25% of the added H_2_S was lost throughout the 10-min exposure (Fig. S1A). This experimental procedure was performed in duplicate with *n*=12 anemones per drug exposure.

We exposed a new subset of anemones (*n*=12 per drug exposure) to decreasing O_2_ levels by bubbling N_2_ gas into the glass containers, each containing anemones in perforated six-well plates. We increased the N_2_ flow rate at 10-min intervals, causing air saturation to decline from approximately 100% to 20% over the 90-min experiment. Anemone responsiveness was recorded immediately prior to each change in N_2_ flow rate. Dissolved O_2_ was continuously monitored and recorded using a FireSting^®^ Optical Oxygen and Temperature meter (PyroScience; FSO2-C4) (Fig. S1B).

We exposed a final subset of anemones (*n*=12 per drug exposure) to gradually increasing temperatures. To ensure sufficiently rapid temperature changes, we reduced the experimental water volume by placing individual anemones into the wells of a 24-well plate, each filled with 2 ml of water and treated with the same drug concentrations as described above. The 24-well plate was then placed into a Fisherbrand™ Incubating Minishaker, and the temperature was increased by 2°C every 10 min. Over the 90-min experiment, water temperature rose from 23°C to 39°C, which we validated via a temperature sensor (Amprobe, TMD-50) within a well that contained 2 ml of water but was otherwise empty. Anemone responsiveness was recorded prior to each 2°C change in temperature.

### Oxidative damage markers

We assessed lipid peroxidation and protein carbonyl content in anemones exposed for 24 h to either 21°C, 31°C or hypoxic conditions. Within each experimental exposure, a subset of anemones was treated with 5 mmol l^−1^ SHAM to inhibit AOX, while another received 1% DMSO to match solvent exposure (*n*=8 per group). Because SHAM is known to inhibit lipoxygenases and peroxidases (Preisig and Kuć, 1987; Romero-Aguilar et al., 2020), any increase in thiobarbituric acid reactive substances (TBARS) and/or protein carbonyls observed after SHAM treatment represents conservative estimates of the AOX-dependent contribution to oxidative damage. Nevertheless, SHAM has been a long-standing and widely used pharmacological tool in studies of AOX in plants and microbes (Romero-Aguilar et al., 2020). Temperature exposures were conducted in Petri dishes (150 ml water, 30‰ salinity) in a Fisherbrand™ incubator to maintain stable conditions. For hypoxia exposures, we bubbled N_2_ into glass chambers (20×12×7 cm; 250 ml, 30‰ salinity) to reach the desired O_2_ level (∼40% air saturation), after which the chambers were sealed. Dissolved O_2_ was continuously monitored using a FireSting^®^ Optical Oxygen and Temperature meter. Following the 24 h exposures, we immediately homogenized individual anemones in approximately 20× volume of ice-cold phosphate buffered saline (PBS; 137 mmol l^−1^ NaCl, 15.2 mmol l^−1^ Na_2_HPO_4_, 2.7 mmol l^−1^ KCl, 1.5 mmol l^−1^ KH_2_PO_4_; pH 7.4). We centrifuged the homogenates for 5 min at 20,000 ***g*** (4°C) and stored supernatants at −80°C until oxidative stress assays were performed. Lipid peroxidation and protein carbonyls were normalized to protein content measured using Bradford (Bio-Rad) and bicinchoninic acid (Sigma-Aldrich) protein assays, respectively, with bovine serum albumin as the standard in both cases.

Lipid peroxidation was assessed using the QuantiChrom™ TBARS Assay Kit (BioAssay Systems, DTBA-100), which quantifies malondialdehyde (MDA), a major TBARS produced during lipid peroxidation. MDA concentration was detected colorimetrically by measuring absorbance at 535 nm and comparing values against a standard curve. Protein carbonyl content was similarly determined using the Protein Carbonyl Content Assay Kit (Sigma-Aldrich). In this assay, protein carbonyl groups are derivatized with 2,4-dinitrophenylhydrazine (DNPH) to form stable dinitrophenyl (DNP) hydrazone adducts, which can be detected spectrophotometrically at 375 nm.

### Statistical analyses

We used a linear mixed-effects model to evaluate the effects of drug exposure (5 mmol l^−1^ SHAM, 5 mmol l^−1^ KCN, 5 mmol l^−1^ SHAM+5 mmol l^−1^ KCN) and experimental treatment (21°C, 31°C, hypoxia) on the O_2_ consumption rate of *N. vectensis*. We included body mass as a continuous fixed effect in the model, and the experimental date as a random effect to account for potential variation across trials. *Post hoc* contrasts within each treatment compared drug exposure groups with the corresponding control using estimated marginal means with Tukey's correction for multiple comparisons. We used logistic regressions with Firth's correction to evaluate whether COX or AOX inhibition altered the responsiveness of *N. vectensis* to increasing H_2_S concentrations, increasing temperature or decreasing O_2_ availability. *Post hoc* contrasts were performed using estimated marginal means with Dunnett's correction to compare the responsiveness of anemones in each drug exposure group to the corresponding control group. Finally, two-way ANOVAs were used to assess the effects of drug exposure (5 mmol l^−1^ SHAM) and experimental treatment (21°C, 31°C, hypoxia) on lipid peroxidation and protein carbonyl content. *Post hoc* comparisons were conducted using estimated marginal means with a Tukey correction only when a significant treatment by exposure interaction was detected. We analysed all data for statistical significance using RStudio (version 2025.05.1+513) with R (version 4.5.1) and visualized the data using GraphPad Prism (v.10.4.2). Results were considered significant at α=0.05.

## RESULTS

**Fig. 2.**
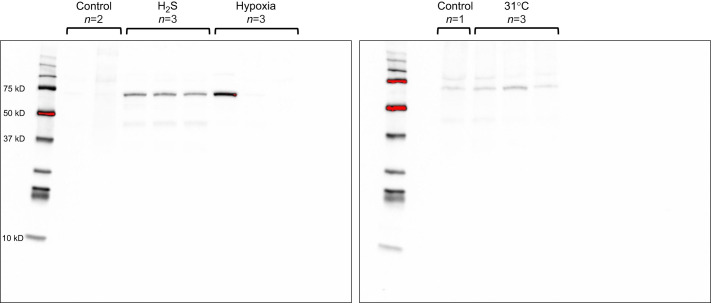
**AOX protein detected using western blots in *N. vectensis* following 24-h exposure to control conditions, elevated H_2_S, low O_2_ and elevated temperature.** *N*=3 per group, run across two gels, with a 10–250 kDa molecular weight ladder.

In *N. vectensis* protein extracts, the AOX antibody cross-reacted with a protein band of ∼70 kDa in size (Fig. 2). Compared with anemones in control conditions, AOX protein levels appeared elevated in all anemone replicates exposed to H_2_S for 24 h. A strong AOX band was detected in one of three individuals exposed to hypoxia. In contrast, AOX expression did not differ noticeably from control in anemones exposed to high temperature (Fig. 2).

**Fig. 3.**
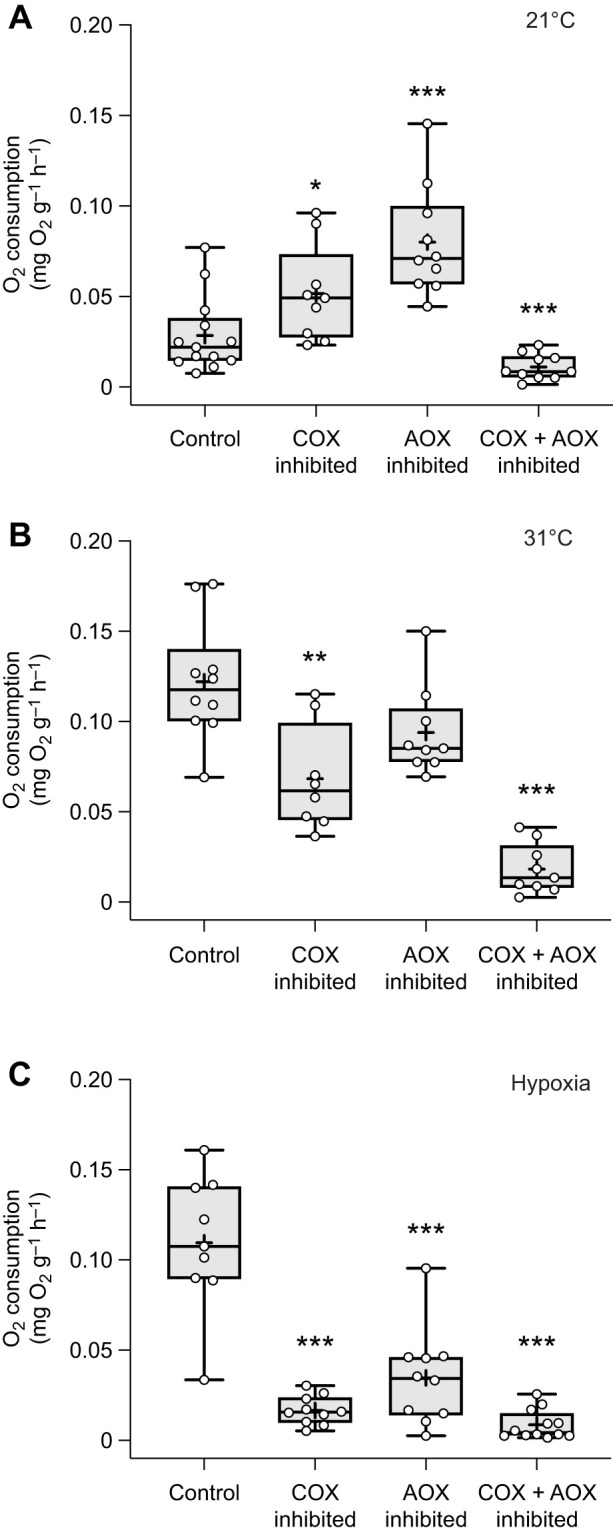
**Oxygen consumption rates of *N. vectensis* under different treatment conditions during exposure to 5 mmol l^−1^ KCN (COX inhibitor), 5 mmol l^−1^ SHAM (AOX inhibitor) or both inhibitors simultaneously.** (A) 21°C, (B) 31°C and (C) hypoxia. Within each treatment condition, significant differences owing to drug exposure are indicated by asterisks relative to the corresponding control (*P<0.05; **P<0.01; ***P<0.001). Box plots show the median (horizontal line), interquartile range (box) and mean (+). Whiskers extend to the minimum and maximum values. *N*=8–13 per group.

We found a significant interaction between drug exposure and experimental treatment on the O_2_ consumption rate of *N. vectensis* (χ²=58.37, *P*<0.001) (Fig. 3). At 21°C, *post hoc* comparisons showed that inhibiting COX or AOX led to a significant increase in O_2_ consumption relative to the control, by 1.8- and 2.8-fold, respectively (COX: *t*=2.34, *P*=0.02; AOX: *t*=4.28, *P*<0.001). At 31°C, COX inhibition reduced O_2_ consumption by 49% (*t*=2.92, *P*=0.004), whereas AOX inhibition had no effect (*t*=0.80, *P*=0.42). Under hypoxic conditions, inhibiting COX or AOX reduced O_2_ consumption by 85% and 73% compared with the control, respectively (COX: *t*=6.05, *P*<0.001; AOX: *t*=5.42, *P*<0.001). In all experimental treatments, simultaneous COX and AOX inhibition reduced O_2_ consumption by at least 60% (21°C: *t*=3.76, *P*=0.003; 31°C: *t*=8.52, *P*<0.001, hypoxia: *t*=10.52, *P*<0.001).

**Fig. 4.**
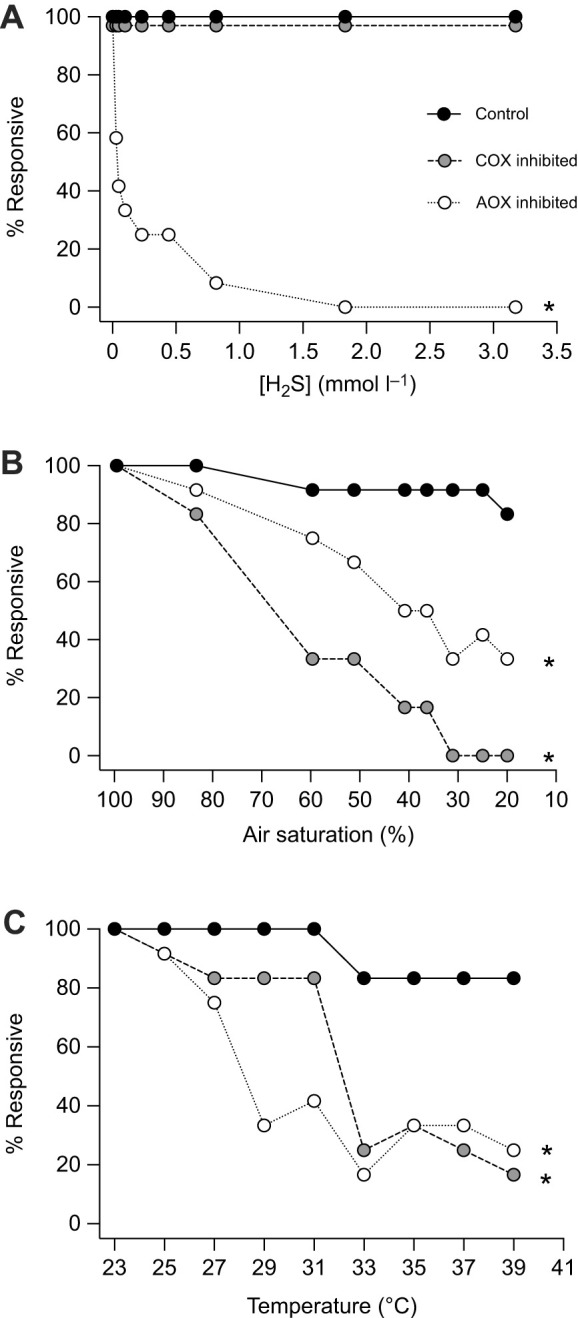
**The proportion of *N. vectensis* that remained responsive while treated with 5 mmol l^−1^ KCN (grey symbols), 5 mmol l^−1^ SHAM (white symbols) or a vehicle control (black symbols) during exposure to different stressors.** (A) Increasing H_2_S concentrations, (B) decreasing air saturation and (C) increasing temperature. Asterisks indicate significant differences in responsiveness curves relative to the control group (*P<0.05). *N*=12 per drug treatment.

In the behavioural sensitivity assays, we found significant main effects of both drug exposure and environmental stressors on anemone responsiveness (Fig. 4). Under increasing H_2_S concentrations, drug exposure (χ²=56.29, *P*<0.001) and H_2_S concentration (χ²=17.65, *P*<0.001) significantly influenced responsiveness, with AOX-inhibited anemones exhibiting significantly different behaviour than control anemones (*z*=5.15, *P*<0.001). Under decreasing O_2_ availability, both drug exposure (χ²=105.92, *P*<0.001) and air saturation (χ²=14.38, *P*<0.001) had significant effects on responsiveness, with both COX- and AOX-inhibited groups differing from the control (COX: *z*=4.04, *P*<0.001; AOX: *z*=7.12, *P*<0.001). Under increasing temperatures, drug exposure (χ²=109.51, *P*<0.001) and temperature (χ²=27.17, *P*<0.001) also influenced responsiveness, with both inhibitor groups again differing from the control (COX: *z*=3.82, *P*=0.001; AOX: *z*=5.02, *P*<0.001).

**Fig. 5.**
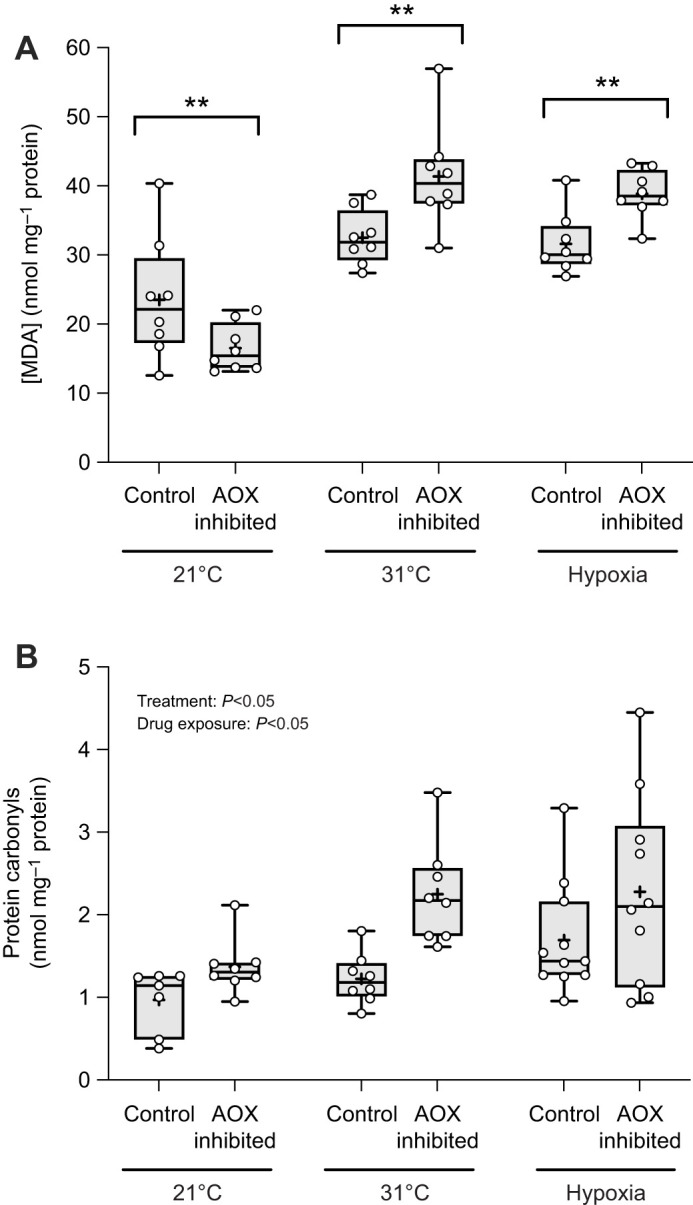
**MDA concentration and protein carbonyl content in *N. vectensis* following a 24-h exposure at 21°C, 31°C or under hypoxia, with treatment of either 5 mmol l^−1^ SHAM (AOX inhibition) or vehicle control.** (A) MDA concentration and (B) protein carbonyl content. A significant treatment by exposure interaction was detected for MDA concentration, with significant differences between treatments within each exposure indicated by asterisks (**P<0.01). For protein carbonyl content, significant main effects of treatment and drug exposure were detected. Box plots display the median (horizontal line), interquartile range (box), mean (+), and minimum and maximum values (whiskers). *N*=7–11 per group.

In the ROS damage assays, there was a significant interaction between experimental treatment and SHAM exposure on lipid peroxidation (*F*=9.38, *P*<0.001). AOX inhibition significantly decreased MDA concentration at 21°C (*t*=2.45, *P*=0.018), but increased it by 27% and 23% at 31°C (*t*=3.11, *P*=0.003) and under hypoxic conditions, respectively (*t*=2.56, *P*=0.014) (Fig. 5A). We also found a significant main effect of drug exposure (*F*=13.242, *P*<0.001) and experimental treatment on protein carbonyl content (*F*=8.23, *P*=0.001). AOX inhibition increased mean protein carbonyl content by 34%, 76% and 37% at 21°C, 31°C and under hypoxic conditions, respectively (Fig. 5B). All data are available in Dataset 1.

## DISCUSSION

In support of our hypothesis, we found that AOX is responsive to H_2_S and promotes tolerance by maintaining respiration despite COX inhibition, even at exceptionally high concentrations. These findings align with other studies linking AOX to H_2_S tolerance in aquatic invertebrates (Wang et al., 2019; Bremer et al., 2021). AOX also appears important for coping with hypoxic and thermal stress, as its inhibition increased sensitivities to these two stressors. In plants, AOX is thought to support hypoxia and thermal tolerance by dampening ROS production and mitigating oxidative damage (Borovik and Grabelnych, 2018; Jayawardhane et al., 2020), a pattern consistent with our findings in *N. vectensis*. Although the role of AOX under stressful conditions is becoming evident in animal models, its importance under relatively benign conditions remains largely unknown. Interestingly, even under relatively benign conditions, AOX influenced the overall cellular oxidative state in *N. vectensis*, suggesting that its function in animals is not limited to stress responses. Taken together, our results suggest that AOX is a critical mechanism of stress tolerance in *N. vectensis* and overlooking its vital role risks underestimating the metabolic flexibility that may underpin the resilience of most animal phyla to a rapidly changing world. More broadly, because eukaryotic life emerged in anoxic and sulphide-rich oceans, AOX may reflect an ancient metabolic strategy that persists in many extant animals, providing valuable insights into the evolutionary trajectory of eukaryotic metabolism (Weaver, 2019).

### Detection of AOX protein in *N. vectensis*

The presence of a putative AOX gene in *N. vectensis* was first reported by McDonald et al. (2009). The predicted AOX is accession number XP_001635929.3 in the protein database at the National Center for Biotechnology Information (www.ncbi.nlm.nih.gov/). Inspection of the previously reported sequence indicates that the glutamate and histidine residues in the iron binding sites are conserved, consistent with a functional AOX protein. Moreover, the sequence contains the C-terminal motif N-P-[YF]-X-P-G-[KQE] that is diagnostic of animal AOX proteins (McDonald et al., 2009). Our western blots using an AOX-specific antibody indicate that the protein is ∼70 kDa in *N. vectensis*, representing the first experimental detection of an AOX protein in a member of the Cnidaria (Fig. 1). Interestingly, the predicted molecular mass based on the animo acid sequence is 37.95 kDa, but this discrepancy in size is likely explained by differences in redox state: oxidized AOX forms disulfide-linked dimers (60–75 kDa), whereas reduced AOX is monomeric (30–39 kDa) (Umbach and Siedow, 1993). In wheat (*Triticum aestivum*), for example, oxidized AOX migrates at ∼70 kDa, whereas the reduced form migrates at ∼36 kDa (Bartoli et al., 2005). The faint band we observed between the 37 and 50 kDa ladder bands in *N. vectensis* may therefore correspond to reduced AOX, although further validation is required.

### AOX function under sulphidic conditions

For most aquatic vertebrates, H_2_S is lethal at low micromolar concentrations, yet many invertebrates exhibit remarkable tolerance (Smith et al., 1976; Gray et al., 2002). Traditionally, this tolerance has been attributed to a high capacity for anaerobiosis (Jahn et al., 1996) and/or the detoxification of H_2_S into more benign forms (e.g. thiosulfate; Oeschger and Vetter, 1992). However, these strategies do not convincingly explain the exceptional tolerance of many invertebrate species, as anaerobic metabolism cannot be sustained indefinitely and H_2_S detoxification rates are generally too low to completely prevent COX inhibition (Grieshaber and Völkel, 1998). Our findings suggest that the AOX pathway may be an important survival mechanism for sulphide-dwelling invertebrates, such as *N. vectensis*. In the field, we found anemones living in tide pool sediments where H_2_S concentrations exceeded the detectible limit of our colorimetric assay (>160 μmol l^−1^ H_2_S; S. Venkatesh, personal observation). Although many fishes perish when exposed to very low H_2_S concentrations (<20 μmol l^−1^; Smith and Osied, 1974), our anemones maintained normal behaviour (responsiveness and an extended tentacle posture) at concentrations more than 100-fold above this level (>3 mmol l^−1^), except the AOX-inhibited group, in which all anemones retracted their tentacles. Remarkably, all anemones survived the H_2_S exposure, including those in which AOX was inhibited. Similar levels of tolerance have been reported in other burrowing marine invertebrates that likely also possess AOX (e.g. the polychaete *Hediste diversicolor*; Gamenick et al., 1996) given its widespread presence across multiple invertebrate lineages (e.g. Annelida; Weaver and McDonald, 2023).

Anemones continued to respire in the presence of cyanide, which, like H_2_S, also inhibits COX. Surprisingly, we found that anemones exhibited higher respiration rates in the presence of KCN compared with control conditions – a phenomenon also observed in some cyanide-resistant plants, yeasts and bacteria (Solomos and Laties, 1976; Akimenko et al., 1983; Veiga et al., 2003a). Unlike the COX pathway, AOX is not tightly coupled to ATP production, so higher O_2_ consumption rates may be required to maintain similar levels of ATP production when COX is non-functional, or to preserve mitochondrial membrane potential (Vanlerberghe, 2013; Veiga et al., 2003b). Paradoxically, AOX inhibition via SHAM had more pronounced stimulatory effects on respiration, despite electron flow being presumably redirected toward the more energy-efficient COX pathway. Although similar findings have been reported in some plant species, the underlying mechanism remains unclear (de Visser and Blacquière, 1984). One possible explanation is that SHAM activates other, potentially non-mitochondrial oxidases that consume O_2_. Indeed, Møller and Berczi (1985) found that the SHAM-stimulated increase in respiration in wheat (*T. aestivum*) roots was accompanied by hydrogen peroxide (H_2_O_2_) production. The addition of catalase suppressed this increase in O_2_ consumption, indicating that the response was driven by H_2_O_2_ formation rather than ATP-coupled respiration (Møller and Berczi, 1985). Further experimentation is required to determine what mechanisms drive the increased respiration observed in SHAM-treated anemones, but this phenomenon represents an interesting avenue for future work. Taken together, AOX is widespread among aquatic invertebrates (Weaver and McDonald, 2023), and our findings highlight AOX as a critical, yet underappreciated, mechanism enabling a sulphide-tolerant lifestyle.

### AOX function under hypoxic stress

Hypoxic conditions are becoming more prevalent in aquatic ecosystems and often promote H_2_S production through anaerobic bacterial respiration. Although these stressors frequently co-occur in marine sediments, we examined the role of AOX in *N. vectensis* under each condition independently to gain more targeted insights into its function. This approach was motivated by our hypothesis that AOX confers stress tolerance via distinct mechanisms depending on the stressor. Our findings show that AOX may support hypoxia tolerance in *N. vectensis*. When AOX was inhibited, anemones became more sensitive to declining O_2_ levels, with only 33% remaining responsive at the lowest O_2_ concentration compared with 83% of untreated individuals. From an ecological perspective, this loss of responsiveness is comparable to an inability to perform essential behaviours in the wild, such as feeding or predator avoidance. Not surprisingly, COX inhibition made anemones even more sensitive to low O_2_ conditions, with a lack of responsiveness from all individuals at the lowest O_2_ level. This response highlights the importance of the COX pathway under hypoxic conditions, likely because it produces more ATP per molecule of O_2_, which is particularly important when O_2_ availability is limited (Jayawardhane et al., 2020). Our respirometry data further support the idea that AOX and COX are each critical for this animal's strong hypoxia tolerance, as inhibiting either enzyme significantly reduced O_2_ consumption with no evident compensation by the other.

AOX has been hypothesized to help maintain cellular redox balance under hypoxia, but empirical support remains limited (Abele et al., 2007). During hypoxia, slowed electron transport can over-reduce the ubiquinone pool in the electron transport chain, increasing the potential for ROS formation via electron leaks at complexes I and III (Solaini et al., 2010). Re-oxygenation following hypoxia can further trigger a ROS burst, similar to that seen in reperfusion–ischemia injury (Storey, 1996). By providing an alternative electron sink, AOX helps prevent the over-reduction of the electron transport chain, thereby limiting ROS production (Vanlerberghe, 2013). In our study, inhibiting AOX in anemones under hypoxia led to higher levels of lipid peroxidation and protein carbonylation, lending support to this hypothesis. Importantly, because anemones were briefly reoxygenated (<60 s) immediately prior to homogenization for oxidative stress assays, a transient ROS burst may have occurred and contributed to the elevated oxidative damage we observed.

We also found that low O_2_ exposure can strongly upregulate AOX protein expression in *N. vectensis*, although responses varied considerably between individuals and our sample size was small. To our knowledge, no studies have directly quantified AOX protein expression in animals under hypoxic conditions. However, several studies in other marine invertebrates have reported hypoxia- or anoxia-induced increases in AOX transcript levels (Sussarellu et al., 2012; Wang et al., 2019; Bremer et al., 2021). Importantly, changes in AOX expression, whether at the transcript or protein level, may not necessarily parallel changes in AOX activity. For example, in *C. gigas*, *AOX* mRNA expression increased within 1 h of hypoxia exposure (Sussarellu et al., 2012), but a measurable increase in electron flow through AOX was only observed 1 h after re-oxygenation (Sussarellu et al., 2013). Such discrepancies may reflect a preparatory response for the ROS burst that often accompanies re-oxygenation events or additional regulatory layers, such as post-translational modifications (Li and Jackson, 2002). In plants, for instance, thioredoxins activate AOX by reducing the disulfide bond between its subunits (Gelhaye et al., 2004). Similar mechanisms have not been investigated in animals. Overall, these findings highlight the need for functional studies to elucidate the role of AOX in supporting hypoxia tolerance, with our results suggesting that AOX helps buffer oxidative damage in *N. vectensis* during hypoxic insults, potentially enhancing resilience to low or fluctuating O_2_ conditions.

### AOX function under thermal stress

Aquatic animals are facing rising water temperatures, and those inhabiting shallow, nearshore habitats are particularly vulnerable (Blewett et al., 2022). In the tide pools where our collections were made in 2024, water temperatures approached 27°C (S. Venkatesh, personal observation) and likely rise much higher at times, underscoring the importance of evaluating the mechanistic basis of thermal tolerance in *N. vectensis*. Our findings suggest that AOX supports thermal tolerance in *N. vectensis*, as AOX inhibition increased sensitivity to elevated water temperatures. Only 20% of AOX-inhibited anemones remained responsive at 39°C, a pattern mirrored by COX inhibition. In contrast, we found differential effects of COX and AOX inhibition on respiration at 31°C, in which AOX inhibition did not significantly change O_2_ consumption rates, whereas COX inhibition reduced rates by ∼50%. In cyanide-resistant plants, the opposite pattern is often observed, with SHAM producing stronger inhibitory effects on respiration than KCN, even across a broad range of temperatures. For example, in soybean (*Glycine max*) leaves, KCN caused only a slight decline in respiration (<10%), whereas SHAM led to a substantially larger reduction (>35%) across temperatures from 10 to 25°C (Atkin et al., 2002). These comparisons highlight that the relative contributions of AOX and COX to respiration under thermal stress may vary markedly across taxa, emphasizing the need for additional animal studies.

As with hypoxia, buffering against oxidative damage has been proposed as a key mechanism by which AOX supports thermal tolerance at thermal extremes (Jayawardhane et al., 2020; Jacobs and Ballard, 2022). Accordingly, we found that AOX inhibition significantly increased lipid peroxidation and protein carbonylation in *N. vectensis* following exposure to 31°C. Among untreated anemones, we found a higher protein carbonyl content under heat and hypoxic stress, suggesting that oxidative damage accumulates under these conditions at a rate that outpaces the capacity for AOX (and other antioxidant defenses) to mitigate ROS production. Surprisingly, at 21°C, AOX-inhibited anemones exhibited higher levels of protein carbonyls, whereas lipid peroxidation showed the opposite trend. The reason for this discrepancy is unclear but may reflect differences between lipids and proteins in their susceptibility, localization and repair mechanisms (Davies, 2000). Very few studies have examined the role of AOX in animals under heat stress, possibly because AOX has received more attention in the context of cold tolerance owing to its potential thermogenic effects (Jacobs and Ballard, 2022). However, Tward et al. (2019) reported that the copepod *T. californicus* markedly increased AOX protein expression following 24-h exposures to both low and high temperatures. In contrast, we observed no change in AOX expression in *N. vectensis* exposed to high temperatures for 24 h. Taken together, our results indicate that although the AOX pathway may dampen ROS production and mitigate oxidative damage in *N. vectensis*, its utility may be constrained by the fact that it limits the ATP production needed to meet the elevated energetic demands of high temperatures – a trade-off that AOX-expressing animals must navigate in a warming climate.

### Perspectives

The AOX mitochondrial pathway is present in most kingdoms of life, yet animal mitochondrial research has been dominated by the ‘conventional’ AOX-lacking electron transport system of vertebrates. This anthropocentric focus has led to an underestimation of the metabolic flexibility in most living organisms. Our findings show that AOX plays a central role in enabling *N. vectensis* to tolerate otherwise toxic sulphides, debilitating hypoxia and noxious temperature. AOX has also been reported in connection with other stressors in *N. vectensis* (e.g. UV radiation, crude oil; Tarrant et al., 2018), broadening its functional significance beyond the conditions examined here. This metabolic flexibility likely facilitates the persistence of species in challenging environments, and in the case of *N. vectensis*, may even enhance invasion potential and underpin the colonization of novel habitats (Reitzel et al., 2008b; Darling et al., 2009). In the context of global climate change, AOX may represent a key player in shaping the distributions of animals across increasingly variable and stressful landscapes.

From a biomedical perspective, *N. vectensis* has emerged as a valuable model for studies on regeneration and wound healing, with the ultimate goal of providing insights into human disease (Röttinger, 2021). Medical models are typically chosen assuming that pathways of interest are evolutionarily conserved, but the presence of AOX – which is absent in humans – may complicate this dynamic. Understanding fundamental differences in metabolic pathways provides crucial context for effectively leveraging AOX-possessing animals in biomedical research. In future, establishing AOX knockout lines in *N. vectensis* would be particularly informative for distinguishing AOX-dependent responses from those that occur in its absence. For *N. vectensis*, this distinction may be particularly important in the sense that regeneration/wound healing and AOX activity may be intrinsically linked through their relationships to ROS balance. Indeed, studies from plants suggest critical roles for AOX in growth and regenerative processes, such as callus formation and organogenesis (Arnholdt-Schmit et al., 2021). Finally, from an evolutionary perspective, eukaryotic life emerged in anoxic and sulphidic oceans, which likely selected for metabolic pathways capable of tolerating or bypassing the effects of H_2_S and low O_2_ (Olson and Straub, 2016). In animals that have retained AOX, this pathway may serve as a molecular imprint of these ancient environmental pressures, reflecting adaptations to both H_2_S exposure and low O_2_ conditions in early animal metabolism (Weaver, 2019). As a cnidarian, *N. vectensis* belongs to one of the earliest diverging animal lineages, potentially retaining features of early animal evolution. Understanding AOX function in extant species such as *N. vectensis*, and why it was lost in others, may provide valuable insights into the trade-offs associated with AOX and evolutionary origins of metabolic flexibility that shaped animal life as we know it.

## Supplementary Material

10.1242/jexbio.252244_sup1 Supplementary information

Dataset 1.
