# Supplementary material for: An ancient oxidase lost in vertebrates promotes extreme stress tolerance in an emerging cnidarian model for ecology, evolution and biomedicine
Source: J Exp Biol. 2026 May 26;229(10):jeb252244. doi: 10.1242/jeb.252244 (PMC13286374; doi:10.1242/jeb.252244)
Supplement: Supplementary information [file jexbio-229-252244-s1.pdf]

Dataset 1.

Available for download at
https://journals.biologists.com/jeb/article-lookup/doi/10.1242/jeb.252244#supplementary-data

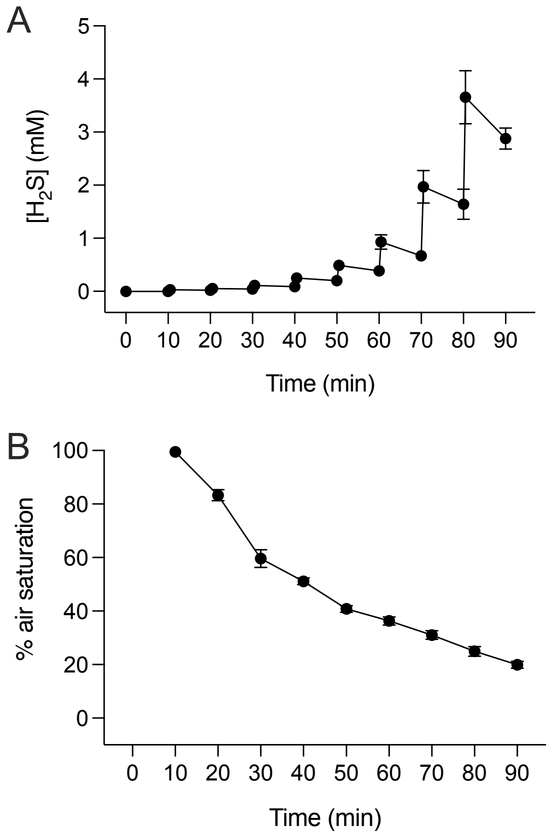

Fig. S1. Changes in environmental stressors during behavioural assays used to assess whether COX or AOX inhibition in *Nematostella vectensis* influences stressor sensitivity. **(A)** H_2S concentrations measured at the start and end of each 10-minute interval during the 90-minute experiment, reflecting both the stepwise increase in H_2S and minor losses due to oxidation and/or volatilization. **(B)** Percent air saturation during the 90-minute hypoxia experiment showing the progressive decline in O_2 across 10-minute intervals.

Journal of Experimental Biology • Supplementary information
